# How to Work with Electromyography Decomposition in Practical Classes of Exercise Physiology and Biomechanics

**DOI:** 10.3390/life12040483

**Published:** 2022-03-26

**Authors:** Jose I. Priego-Quesada, Márcio F. Goethel, Klaus Magno Becker, Ricardo J. Fernandes, João Paulo Vilas-Boas

**Affiliations:** 1 Research Group in Sport Biomechanics, Department of Physical Education and Sports, University of Valencia, 46010 Valencia, Spain; 2 Research Group in Medical Physics (GIFIME), Department of Physiology, University of Valencia, 46010 Valencia, Spain; 3 Porto Biomechanics Laboratory (LABIOMEP-UP), University of Porto, 4000-008 Porto, Portugal; gbiomech@fade.up.pt (M.F.G.); klausmagnobecker@gmail.com (K.M.B.); ricfer@fade.up.pt (R.J.F.); jpvb@fade.up.pt (J.P.V.-B.); 4 Centre of Research, Education, Innovation, and Intervention in Sport, Faculty of Sport, University of Porto, 4000-008 Porto, Portugal

**Keywords:** EMG, muscle, neuromuscular activation, fatigue, motor units

## Abstract

Concepts about motor unit recruitment are important learning contents in exercise physiology and biomechanics classes that are usually taught theoretically. In the last few years, great advances have occurred in the decomposition of surface electromyography, allowing the learning of theoretical contents in an experimental way. In this tutorial paper, we have described the decomposition of surface electromyography methodological aspects and examples to teach motor unit recruitment concepts in exercise physiology and biomechanics practical lessons. This work has the aim to facilitate physiology and biomechanics academics to introduce this technique in practical classes.

## 1. Introduction

**Figure 1:**
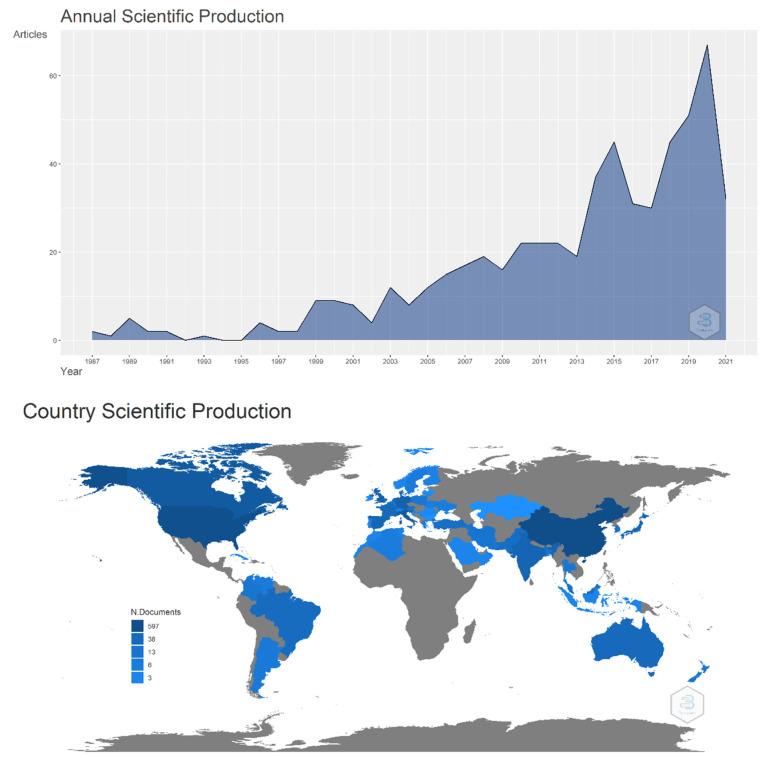
Annual scientific production and country scientific production. These graphs were obtained with the Bibliometrix package in RStudio, with the data obtained by the search in Scopus database using the keywords “decomposition” AND “surface” AND “electromyography”.

Concepts about motor unit recruitment are important learning contents in exercise physiology and biomechanics classes that are usually taught theoretically or through simulation software applications [1,2,3]. These teaching solutions are usually carried out due to their logistical easiness and non-invasive nature (compared to practical lessons that were carried out a few decades ago with animal models) [4,5]. However, in the last few years, great advances have occurred in the decomposition of surface electromyography (D-sEMG), allowing the learning of theoretical contents in an experimental way. Although the discriminated access to this technology is a major limitation, the knowledge and education of the academics responsible for those practical classes might also be an obstacle [6]. However, this technique is also expanding through universities worldwide, as Figure 1 shows the high increase of papers published in recent years using this technique and a world map of the location of these publications. In this paper we have described D-sEMG methodological aspects and examples to teach motor unit recruitment concepts in exercise physiology and biomechanics practical lessons.

## 2. Basis of Motor Unit Recruitment and Its Assessment

From the concept that movement generation starts with a neural signal transmitted through axonal action potentials to one or different motor units [6,7], different definitions and theoretical contents should be presented during physiological and biomechanical classes. A motoneuron refers to the neuron in the central nervous system that extends its axon into a muscle or gland, and a motor unit is the number of muscle fibers that are innervated by the same motoneuron [8]. The number of motor units activated and their corresponding firing rates determines the muscle force generated and its interaction with the environment, providing information about the individual control of the neuromuscular system [6,9,10]. Since 1957, when experiments were carried out with anesthetized cats [11], it has been commonly accepted that motor units are recruited in an increasing size order [9].

However, the specific knowledge about the motor unit types and respective firing rates has undergone great changes through the recent decades, in part due to methodological limitations. Until the 1990s, the Analytic Hierarchy Process theory was commonly accepted, which states that the smaller motoneurons generate lower firing rates, with a lower threshold and wider and smaller amplitude force twitches than the larger ones [9]. However, posterior studies support the Onion-Skin scheme theory formulating that the first recruited motor units have higher firing rates than later recruited ones [9,12]. Therefore, lower-threshold motor units are able to sustain force production for relatively longer time periods (with higher firing rates), while the higher-threshold and higher-force-producing motor units are not activated at low-force levels and remain available to contribute to higher-force levels, despite low firing rate levels [9].

The motor unit recruitment assessment during exercise and in different populations is of great interest since it can provide relevant information about physical capacity, the composition/function of different muscles and the effect of a clinical intervention [10,13,14,15,16,17,18]. Moreover, its assessment is useful in providing information about standing balance [19], force levels during painful contractions [20] and lower limb mobility and strength [21]. Motor unit action potentials result in electric signals. Although they can be assessed using electromyography intramuscular electrodes, their invasive nature and small volume detection have resulted in more surface electromyography (sEMG) related studies [18,22] and their exclusion in teaching practical lessons. Motor units’ recruitment has been assessed using sEMG by frequency and spectral analysis [13,14,15], but this type of analysis has been criticized due to a difficult association between fiber type and conduction velocity [23]. The emergence and advances of D-sEMG has provided an interesting method to assess motor units’ recruitment and transfer their use to physiology and biomechanics practical lessons.

## 3. Decomposition of Surface Electromyography and Methodological Aspects for Practical Lessons

**Figure 2:**
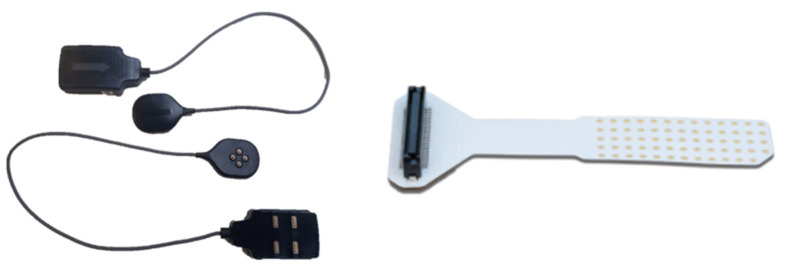
Different sensors used for the decomposition of surface electromyography: Trigno Galileo Sensor of Delsys company and GR04MM1305 of Bioelettronica (**left** and **right** panels, respectively). The Bioelettronica sensor image was obtained from the company website (https://otbioelettronica.it/, accessed date: 15 July 2021).

Since D-sEMG is spreading through academia and will be (sooner or later) as widely used as conventional sEMG, we aimed to facilitate physiology and biomechanics academics to introduce this technique in practical classes. It is important to highlight that this method requires expertise in signal acquisition, analysis and interpretation, particularly for research purposes [7]. There are different brands and models of D-sEMG technology (each with its peculiarities), which is the reason why teachers should know the selected system specifications and technical aspects well. The current manuscript focuses on the Delsys^®^ system because it is used at our lab and is one of the historical leaders in the field (NeuroMuscular Research Center of Boston University) [9,24,25]. It uses the 23 × 30 mm Trigno Galileo Sensor (Delsys, Inc., Natick, MA, USA) with four electrodes (5 mm of inter-electrode distance), 19 g of mass and 2222 Hz of the maximum sampling rate (Figure 2, left panel). Another example of a frequently used instrument is the high-density electrode grid located around the body region of interest [8,26], such as the sensor of Bioelettronica^®^, consisting of a matrix of 8 × 8 concentric electrodes with a 10 mm diameter and 10 mm inter-electrode distance [26] (Figure 2, right panel).

**Before data acquisition**, the skin and sensor placements should respect sEMG guidelines [27,28] and the skin should be prepared by shaving and cleaning the area of interest with isopropyl alcohol [28]. Delsys suggested that, when using their equipment, it is not necessary to shave the skin, which is an advantage in practical lessons when students are instrumented. Moreover, the SENIAM guidelines are recommended for the sensors’ placement on the muscles [27,28], particularly focusing on setting the electrodes over the muscle belly and parallel with the muscle fiber orientation. In the case of vastus lateralis, which is the muscle to be evaluated by the examples proposed in this document, electrodes should be placed at 2/3 on the line from the anterior spina iliaca, superior to the lateral side of the patella, in the direction of the muscle fibers [29]. Finally, although for the Galileo sensor it is not necessary, other D-sEMG sensors may be required to adjust the inter-electrode distance that usually ranges from 3–10 mm, depending on practical criteria such as the muscle size [7].

One of the main aspects to consider when selecting the exercises to be carried out in the practical classes is the very long signal processing time. Therefore, regardless of the chosen exercise, short recordings (no more than 2 min and, preferably, less than 1 min) should be made to reduce this processing time. Moreover, a previous and final rest in the recording process is mandatory to improve the motor unit’s identification. Finally, even if the system has been working properly only with isometric contractions [24], it also works currently with cyclical dynamic exercises.

**Figure 3:**
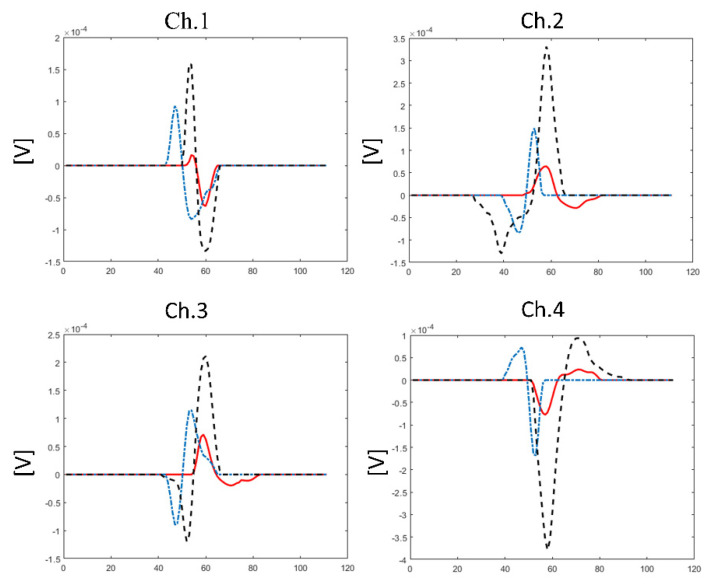
Graphical visualization of motor units’ identification using selective amplitude, duration and inter-pulse interval criteria. Three different motor units were represented in order of their threshold activation: motor unit number 1, 10 and 20 (continuous, dashed with dots and dashed lines, respectively). This graph can be used to evidence to the students how the algorithm works and the signal differences between motor units in the four channels of the sensor.

**After registering** the signal with the amplification and filters (20–450 Hz), made available by the Delsys software, it is necessary to export the datafile, importing it to a specific software to perform the motor unit’s identification. Delsys uses the Precision Decomposition III algorithm (version 1.1, Delsys, Inc., Natick, MA, USA) that uses artificial intelligence to identify motor units using selective amplitude, duration and inter-pulse interval criteria [24,30] (Figure 3). As we have commented during the selection of the exercises, the processing time is long, and the algorithm only works with files with short durations. One possible proposal is to carry out this practical class in two sessions on different days, the first session being used for the students to perform the exercises and record the signals, and the second session to interpret the graphs obtained.

## 4. Examples of Practical Lessons Using Decomposition of Surface Electromyography for Teaching Motor Units’ Recruitment

In this section, we are going to show different types of practical lessons’ proposals that teachers can select or, even, create new approaches based on these ideas. We have decided to base all the proposals in the assessment on the vastus lateralis muscle for different reasons. Vastus lateralis has the main function of knee extension, which makes it an important contributor in movements that are affordable and easy to evaluate (such as squats or jumps). As it is the largest muscle of the quadriceps, it is easier for the students to localize and instrument with sEMG sensors. Moreover, it is composed by a considerable proportion of type II fibers (58%; [31]), making it more attractive to assess the different recruited motor units. Finally, there is extensive literature assessing vastus lateralis’ neuromuscular activity, probably due to its relation with patellofemoral pain and knee osteoarthritis [32,33,34], making it easier to propose activities for the students (e.g., comparing results obtained in the practical lessons with those reported in the scientific literature).

The instrumentation and data collection of the following examples can be summed up in the following points:

- Data were collected using the Trigno Galileo Sensor (Delsys, Inc., Natick, MA, USA).
- Electrodes were placed according to the SENIAM guidelines [27,28]. Therefore, the electrodes were placed at 2/3 on the line from the anterior spina iliaca, superior to the lateral side of the patella, in the direction of the muscle fibers. For the determination of its location, the assessed person was sitting on a table with their knees in slight flexion and the upper body slightly bend backward.
- Data were recorded with a sampling rate of 2000 Hz.
- Data processing was performed by the software of Delsys that uses the Precision Decomposition III algorithm (version 1.1, Delsys, Inc., Natick, MA, USA).

**Figure 4:**
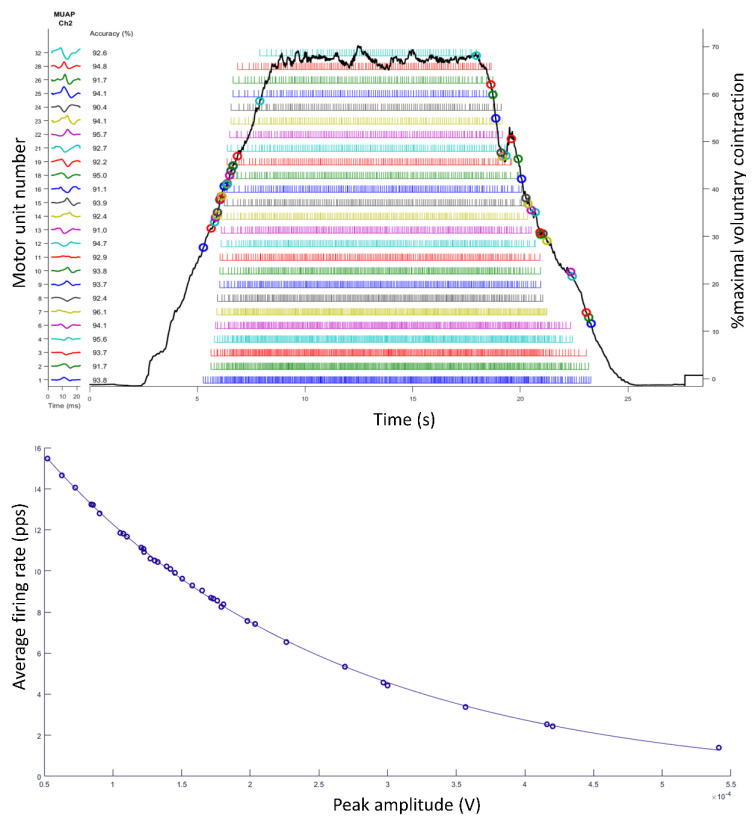
Isometric knee extension at 70% of maximal voluntary contraction. At the **upper** panel, the solid line represents a trapezoid shaped force traced over time, the segmented lines exemplify each motor unit and their respective firing rates, and circles indicate their onset and offset recruitment and de-recruitment thresholds. The relationship between the peak amplitude (x-axis) and average firing rate (y-axis) of each motor unit is displayed in the **bottom** panel.

**Isometric contraction.** The first approach that we propose is to assess an isometric contraction of the vastus lateralis. The upper panel of Figure 4 shows how muscle recruitment occurs. To reach 70% of the maximum voluntary contraction, motor units are recruited in an increasing amplitude order and decreasing firing rate order (see Figure 4, bottom panel, showing that the greater amplitude motor units have a lower firing rate). This approximation and these graphs are very useful to practically explain the Onion-Skin scheme theory [9,12].

**Figure 5:**
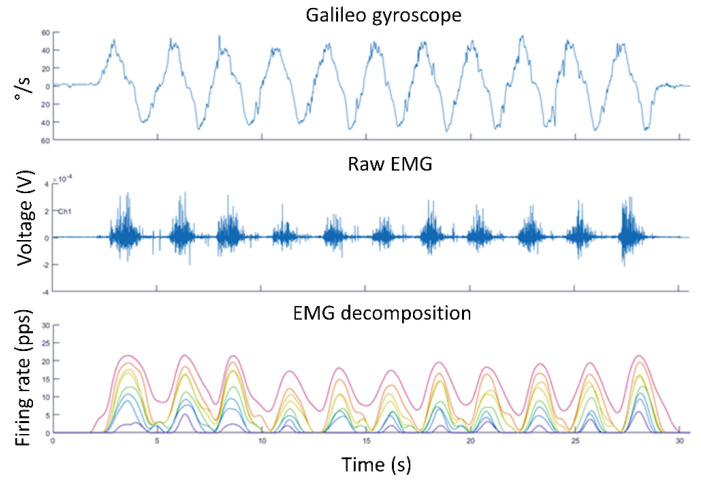
Synchronized data during squat repetitions.

**Dynamic contraction—squat exercise.** The next proposal aims to show the motor unit activation during dynamic exercises (such as a squat). In the Figure 5 upper panel, the Galileo sensor built-in gyroscope data that allow the synchronization of the movement kinematics with the muscle electric signal can be observed. The decomposition process is also displayed; how, from the typical raw sEMG signal (middle panel), the decomposition process makes it possible to visualize the different motor units’ behavior and how they are recruited during exercise, synchronously with the movement (bottom panel). As the exercise performed was of a low force demand, very stable motor units’ behavior is observable during the different repetitions that can be contrasted with the following practical activity (maximal dynamic contraction).

**Figure 6:**
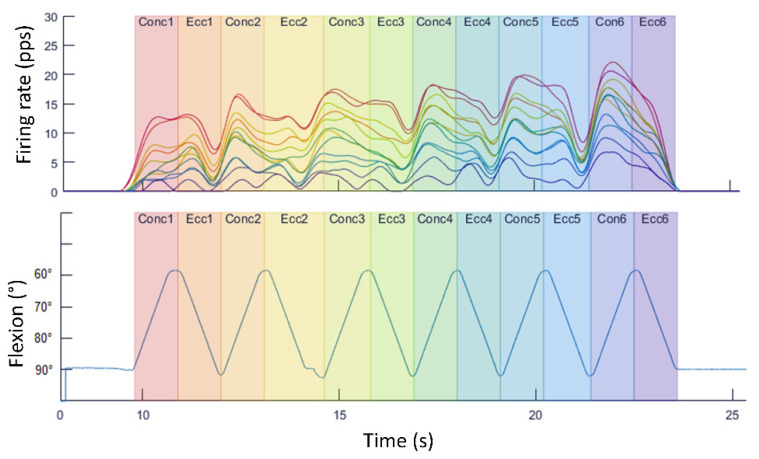
Decomposition of the electromyography signal and movement data during maximum knee extension/flexion repetitions in an isokinetic dynamometer.

**Maximal dynamic contraction—knee extensions/flexions using isokinetic dynamometer**. The next proposal can be useful to show the difference in the motor recruitment behavior with the previous example. In this case, our proposal consisted in that a student would perform six repetitions of knee extensions/flexions at maximum force during concentric and eccentric contractions using an isokinetic dynamometer. The torque necessary for this exercise requires a greater demand from the performer (compared to the squats), resulting in a completely different motor unit behavior. In fact, while in the squat exercise, a very stable behavior could be observed (Figure 5), the isokinetic series shows how the units’ firing rates increase as the repetitions progress (Figure 6). This adaptation was observed by several studies that evidenced that an increase in the motor unit’s excitation is a mechanism to maintain muscle force during a demanding task [25,35,36].

**Figure 7:**
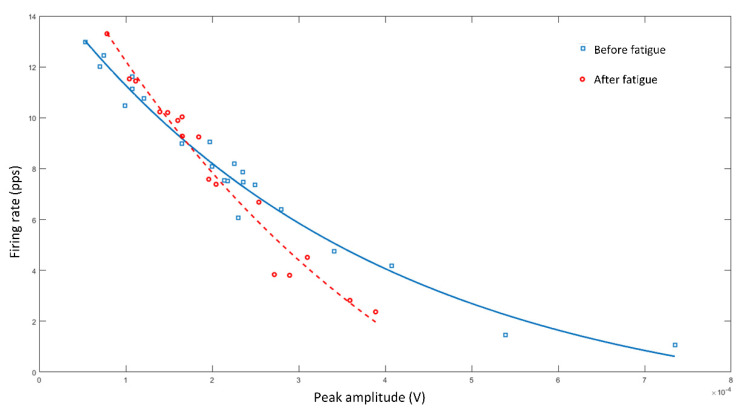
Relationship between the peak amplitude and average firing rate (x and y axes, respectively) of the different motor units recruited during knee flexions before and after fatigue (solid line with square points and segmented line with circles points, respectively). Both curves have an R2 of 0.97.

**Effect of fatigue on maximal dynamic contraction.** Maintaining the isokinetic dynamometer approach, motor units’ recruitment could be analyzed pre and post a fatigue inducing protocol. Fatigue can be implemented from different ways proposed in the literature, such as repeating dynamic or isometric contractions [34,35,37], or jumps (e.g., squat or drop jumps) [38,39]. The advantage of the latter strategy is the possibility to generate fatigue in several students simultaneously, being less time consuming. To observe the fatigue effect on motor unit recruitment, the relationship between the firing rate and the motor unit amplitude can be presented (Figure 7), evidencing how fatigue reduces the threshold of the low firing rate and high amplitude motor units (with no clear effect on the high firing rate motor units). This decrease in the recruitment threshold is well reported [34,35,40] and this experience would allow to empirically explain how lower-threshold motor units are considered resistance fiber units, while higher-threshold motor units produce higher force levels but are more fatigable [41]. Finally, it is important to mention that each person could present a particular degree of fatigue-induced changes in motor unit recruitment behavior [35].

## 5. Conclusions

The decomposition of surface electromyography is an excellent methodology to describe motor recruitment concepts in an experimental lesson. Although it is important to be an expert for its use in the research field, the current manuscript offers some practical and graphic proposals. These are directly available through commercial software and can be implemented at exercise physiology and/or biomechanics practical lessons. These proposals can support students when learning complex physiological and biomechanical concepts that are challenging in the traditional theoretical course format. Furthermore, it allows students and teachers to update new theories of motor unit recruitment, discuss concepts and propose activities to compare/discuss data from the specialized literature. This methodology might also facilitate the knowledge translation from the classroom to daily exercise, rehabilitation, and research applications.
